# Neuropsychological predictors of conversion from mild cognitive impairment to dementia at different timepoints

**DOI:** 10.1002/brb3.3098

**Published:** 2023-08-07

**Authors:** Davide Quaranta, Naike Caraglia, Federica L'Abbate, Guido Maria Giuffrè, Valeria Guglielmi, Giovanna Masone Iacobucci, Paolo Maria Rossini, Paolo Calabresi, Camillo Marra

**Affiliations:** ^1^ Neurology Unit Foundation Policlinico Agostino Gemelli IRCCS Rome Italy; ^2^ Department of Neuroscience Catholic University of Sacred Heart Rome Italy; ^3^ Clinical Psychology Unit Foundation Policlinico Agostino Gemelli IRCCS Rome Italy; ^4^ Brain Connectivity Laboratory, Department of Neuroscience and Neurorehabilitation IRCCS San Raffaele Roma Rome Italy; ^5^ Memory Clinic Foundation Policlinico Agostino Gemelli IRCCS Rome Italy

**Keywords:** Alzheimer's disease, episodic memory and semantic memory, mild cognitive impairment

## Abstract

**Introduction:**

: Impairment of episodic memory is largely considered the main cognitive marker of prodromic Alzheimer's disease (AD). Nevertheless, the neuropathological process in AD starts several years before and, apart from biomarkers well defined in the Amyloid (A), Tauopathy (T), Neurodegeneration (N) framework, early clinical and neuropsychological markers able to detect mild cognitive impairment (MCI) due to AD before the appearance of memory disorders are lacking in clinical practice. Investigations on semantic memory have shown promising results in providing an earlier marker of dementia in MCI patients.

**Methods:**

: A total of 253 MCI subjects were followed up every 6 months for 6 years—186 converted to dementia and 67 remained stable at the sixth year of follow‐up. Twenty‐seven patients progressed in the first 2 years (fast converters), 107 in the third to fourth year (intermediate converters), and 51 after the fourth year of follow‐up (slow converters).

**Results:**

: Stable MCI subjects performed better than fast decliners in Mini–Mental State Examination (MMSE), several long‐term memory scores, and category verbal fluency test (CFT); stable and intermediate converters differ only in MMSE and CFT tests; and stable and slow converters differ only in MMSE and phonological/semantic discrepancy score.

**Conclusion:**

: Early impairment of semantic memory could predict the evolution to AD before the onset of episodic memory disorders, and the discrepancy between phonological and semantic verbal fluency could be able to detect this impairment in advance in respect of simple CFT tests. The assessment of different aspects of semantic memory and its degradation could represent an early cognitive marker to intercept MCI due to AD in clinical practice.

## Graphical abstract

Semantic memory is an earlier neuropsychological marker than episodic memory in detecting MCI due to AD. Semantic/phonological discrepancy scores are the most accurate index of early semantic impairment in MCI subjects.

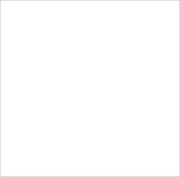

## 1 INTRODUCTION

The construct of mild cognitive impairment (MCI) has received great attention since its first formulation (Petersen et al., 1999). When presenting, this condition is the main predictor of overt dementia in the successive few years. Several epidemiological studies have shown that from a former population of MCI subjects, only roughly 50% will present dementia in the following 10 years, while others will remain in a stable condition of MCI or even return to normality. The recent promising (yet not definitive) progress in the development of disease‐modifying therapies for Alzheimer's disease (AD), picked in the accelerated FDA authorization for the use of anti‐beta‐amyloid monoclonal antibodies, will require the use of reliable markers able to early identify, among individuals with MCI, those already in a prodromal stage of AD. Early diagnosis of prodromal AD can currently be obtained by combining a variety of imaging (FDG‐PET, MRI) and tests based on disease biomarkers (amyloid‐PET or CSF). However, most of these tests are characterized by high cost, low availability considering the estimated population with MCI, and lastly a potential risk related to the invasiveness of the CSF analysis. In conclusion, the number of individuals who would access such diagnostic procedures will be potentially enormous resulting in predictable unsustainable costs to public health systems. However, apart from the perspectives connected to potential disease‐modifying therapies, early diagnosis of dementia may favor the development of coping strategies in caregivers (de Vugt & Verhey, 2013) and also be advantageous from the economic point of view (Barnett et al., 2014; Getsios et al., 2012).

For all of these reasons, the identification of less expensive and larger available neuropsychological markers could be crucial in the detection of those MCI subjects who will be candidates for more expensive and invasive investigations.

Since its first definitions (Petersen et al., 1999; Winblad et al., 2004), the impairment of episodic long‐term memory has been considered the most relevant feature of MCI, being successively included in the National Institue of Aging (NIA) ‐ Alzheimer's Association (AA) criteria for the diagnosis of MCI due to AD (Albert et al., 2011) and in the International Working Group (IWG) recommendations to detect the onset in the prodromal phase of typical AD (Dubois et al., 2014, 2016). Several studies have reported performances on episodic memory tasks (delayed recall and cued recall in particular) to be the main predictors of progression to AD (Fleisher et al., 2007; Gainotti et al., 2014; Sarazin et al., 2007), particularly when more than one memory test is impaired (Loewenstein et al., 2009; Perri et al., 2007) or composite scores are used (Marra et al., 2015; Piccininni et al., 2020).

In recent years, very early changes involving language and semantic memory have been observed in MCI, suggesting that they could be potential markers for the early detection of subjects who will convert to dementia as well. It has been proposed that deficits in context‐free memory, which includes semantic memory, may actually represent one of the earliest clinical markers of AD (Didic et al., 2011), based on the fact that in the early transentorhinal stage of the disease, neurofibrillary tangles related to tau pathology are mainly present in the subhippocampal region (perirhinal and entorhinal cortices). This region, which is functionally integrated into an anterior mesiotemporal network, has been shown to play a key role in context‐free memory (Davies et al., 2004; Didic et al., 2011). As a consequence, changes in some aspects of semantic memory may occur very early in AD patients. Typically, semantic memory was assessed using basic neuropsychological tests of language, such as the category fluency test (CFT) (Chasles et al., 2019). There are several versions of tasks assessing category fluency that are based on different semantic categories (e.g., animals [Morris et al., 1989]; animals, fruits, and car brands [Novelli et al., 1986]; animals and vegetables [Conca et al., 2022]; birds and articles of furniture [Quaranta et al., 2016]) and which score corresponds to the number of words produced during a given time interval (e.g., 1 min for category). Individuals who later progressed to dementia have been reported to generate fewer words than nonprogressors at baseline evaluation (Amieva et al., 2004; Gallucci et al., 2018). These findings are in agreement with previous studies supporting the diagnostic role of CFT tests in detecting AD, especially when compared to phonological verbal fluency (Henry et al., 2004; Monsch et al., 1992; Quaranta et al., 2016), which is based on phonological lexical retrieval and not on the retrieval of words belonging to a specific semantic category. However, both the phonological and category fluency tasks include several executive aspects (Reverberi et al., 2014). Marra et al. (2021) developed an adjusted score that distinguished the executive component of word retrieval from the purely semantic aspects and it was reported to be reliable in identifying MCI individuals who progress to dementia.

The semantic memory attainment of MCI individuals has been explored also using item‐level analysis to detect qualitative changes in CFT words’ production. Amnesic MCI (aMCI) subjects produce words that are more frequent and acquired earlier (Biundo et al., 2011) and are more typical (Vita et al., 2014) than words produced by normal subjects. The production of highly typical words in aMCI also predicted the conversion to AD after 2 years of follow‐up (Vita et al., 2014). CFT performances of individuals with MCI who will convert to dementia are also characterized by weak semantic relationships (Quaranta et al., 2019) and reduced clustering effect (Haugrud et al., 2011; Quaranta et al., 2019).

Deficits in other cognitive domains, such as executive functions (Duara et al., 2011; Fleisher et al., 2007; Quaranta et al., 2014; Tabert, 2006) and visuospatial abilities (Amieva et al., 2004; Buchhave et al., 2008; Saunders & Summers, 2011) have been reported as predictors of conversion to AD. In general, the association of memory disturbances with impairment in other cognitive domains, defined as MCI multiple domains, has been linked with a higher risk of progression to dementia (Bermejo‐Pareja et al., 2016; Fischer et al., 2007; Forlenza et al., 2009; Göthlin et al., 2017; Han et al., 2012; Lopez et al., 2007; Michaud et al., 2017; Nordlund et al., 2010; Quaranta et al., 2014). However, this claim is still controversial, since a common operational definition of this condition is still lacking (Göthlin et al., 2017; Jak et al., 2009; Piccininni et al., 2020).

The studies aimed at identifying the neuropsychological markers of MCI progression are generally based on either the prognosis at a specific timepoint (e.g., the risk of conversion after 2 years of follow‐up) or on survival analyses. A potentially relevant aspect that may deserve some attention is the possibility that individuals with MCI who progress early after the baseline could have different features as compared to patients who will convert later and to patients who will not convert to dementia even after a long follow‐up.

This kind of investigation may be of some interest for clinical and theoretical reasons.

From the clinical point of view, it could be useful to stratify the risk of a single subject as a fast or a slow progressor, especially in light of the recent progresses in terms of disease‐modifying therapies.

From the theoretical point of view, it is conceivable that focusing on the severity of episodic memory impairment, we detect only patients very proxy to the progression to AD. On the contrary, linguistic attainment investigations could intercept also those MCI subjects, less impaired in episodic memory, who will progress to AD at a longer distance from the baseline. From this perspective, linguistic abilities could be reckoned as very early markers of disease onset, at least when compared with markers of faster progression.

The purpose of the present study was to assess the possible role of neuropsychological profiles at the baseline in predicting the rate of progression from MCI to overt dementia. This purpose can be viewed as twofold: (1) to distinguish neuropsychological functions impairment predicting a slow or fast rate of progression to dementia and (2) to describe neuropsychological behavior predicting progression at a higher distance from the baseline, which may be useful to detect patients already in an early stage of the disease that will progress lately to AD. Our prediction was that lexical–semantic impairment, which has been recently proposed as a marker of early changes in structure belonging to the medial temporal cortex, may predict progression to dementia before episodic memory in the AD disease course.

## 2 MATERIALS AND METHODS

### 2.1 Participants

Participants were consecutively enrolled among individuals referring to the Neuropsychology Unit of the Agostino Gemelli University Hospital Foundation IRCCS. For the definition of aMCI, the following characteristics were considered: (1) memory impairment (as compared to the past) reported by the subject or an informed caregiver lasting no more than 2 years; (2) evidence of memory deficits on the delayed recall of the Rey Auditory Verbal Learning Test (RAVLT) and/or delayed recall of the Rey–Osterrieth Complex Figure (ROCF); and (3) preserved independence in daily activities (to be performed as usual or with minimal difficulty). All enrolled individuals achieved a Clinical Dementia Rating (CDR) score of 0.5. Efficiency in activities of daily living was assessed by the ADL (Katz et al., 1963) and IADL (Lawton & Brody, 1969) scales. All of the individuals underwent brain MRI and PET‐FDG examination confirming neurodegenerative pathology and excluding other pathologies. According to the clinical, neuropsychological, and imaging investigations, all MCI patients met the criteria for MCI due to AD at an intermediate level of certainty (Albert et al., 2011).

Exclusion criteria were as follows: head trauma, epilepsy, alcoholism, or other major neurologic or psychiatric illness. No patients had medical conditions potentially associated with cognitive impairment (i.e., renal or liver failure, thyroid dysfunction, folate and/or vitamin B12 deficiency). All patients referring to the neuropsychological unit gave their informed consent to use their clinical data in a retrospective anonymized study, according to the guidelines of our ethics committee and following the Declaration of Helsinki.

The sample was formed by 253 (129 women) individuals affected by MCI, with a mean age of 72.95 years (standard deviation [SD] = 6.596), mean literacy of 10.76 years (SD = 4.464), and mean MMSE score of 25.69 (SD = 1.970).

### 2.2 Neuropsychological assessment and follow‐up

Participants were evaluated with a neuropsychological battery including MMSE; RAVLT (Carlesimo et al., 1996); copy and delayed recall of ROCF (Caffarra et al., 2002); phonological verbal fluency (Carlesimo et al., 1996); categorial verbal fluency (Quaranta et al., 2016); copy of figures with and without landmarks (Carlesimo et al., 1996); Raven's Colored Progressive Matrices (Carlesimo et al., 1996); Stroop's test (Caffarra et al., 2002); digit span forward and backward (Monaco et al., 2013); objects naming (Miceli et al., 1994); and the Multiple Features Targets Cancellation (Marra et al., 2013). The semantic–phonological delta (SPD) was computed as proposed in a previous study (Marra et al., 2021). Briefly, it is computed as the difference between categorical and phonological verbal fluency, both divided by the number of stimuli for each task. A negative value corresponds to worse semantic fluency in comparison to phonological fluency.

During the follow‐up, participants with MCI were assessed every 6 months, undergoing a complete neurological and medical examination and a neuropsychological assessment. At each follow‐up visit, the progression to dementia was assessed by a neurologist blinded to the results of the baseline neuropsychological examination. The diagnosis of dementia was formulated if the clinical criteria for dementia due to AD (McKhann et al., 2011) were satisfied, and participants obtained a CDR score of 1. The diagnosis of AD must be confirmed in a following follow‐up visit after 6 months. The follow‐up was carried on for 6 years.

The subjects who progressed to dementia were subdivided into three groups on the basis of the time of conversion: fast (during the first 2 years of follow‐up), intermediate (conversion occurring after 2–4 years of follow‐up), and slow converters (conversion occurring over 4 years from the baseline).

### 2.3 Statistical analysis

Mean comparison between groups was carried out by one‐way ANOVAs followed by the Games–Howell post hoc test. All of the variables that displayed a statistically significant difference between groups were set as dependent variables of a multiple‐variable multinomial logistic regression model. The status category (fast, intermediate, and slow converters) was the dependent variable. A bootstrap procedure, with 1000 iterations, was performed. Odds ratios (OR) were determined to estimate the degree of association between the dependent variables and each category of the status variable, posing the “stable” status as the reference. An OR of >1 indicates that the independent variable is associated with an increased probability to belong to that specific category (the higher the value of the independent variable, the higher the probability [odds] to belong to that category). An OR of <1 indicates that the independent variable is associated with a decreased probability to belong to that specific category (the higher the value of the independent variable, the lower the probability [odds] to belong to that category).

## 3 RESULTS

During the follow‐up period, 186 patients developed overt dementia, and all of them satisfied the clinical criteria for dementia of the Alzheimer's type (McKhann et al., 2011) except one, who received a diagnosis of behavioral variant of frontotemporal dementia during the follow‐up and was therefore excluded from all subsequent analyses.

As shown in Table 1, we found statistically significant differences between the four groups for scores obtained on MMSE; RAVLT immediate recall, delayed recall, and accuracy; phonological and categorical verbal fluency; and SPD.

**TABLE 1:** Comparison of demographic features and neuropsychological scores between subjects who did not progress to dementia during the follow‐up period (stable) and subjects who were classified as fast, intermediate, and slow converters.

	Stable (*N* = 67)		Fast (*N* = 27)		Intermediate (*N* = 107)		Slow (*N* = 51)			
	*M*	*SD*	*M*	*SD*	*M*	*SD*	*M*	*SD*	*F*	*p*
Age	71.4	6.52	74.7	7.01	73.5	6.76	72.9	5.85	2.118	.098
Literacy	10.5	4.53	9.4	4.66	11.2	4.38	10.8	4.43	1.200	.310
MMSE	26.8	1.61	24.4	1.74	25.3	1.82	25.8	2.16	**13.910**	**.000**
RAVLT immediate recall	25.6	6.61	20.4	5.83	23.8	6.38	24.8	5.87	**4.652**	**.003**
RAVLT: delayed recall	2.0	1.66	0.7	1.10	1.6	1.42	2.1	1.68	**6.142**	**.000**
RAVLT: accuracy	0.8	0.12	0.7	0.12	0.7	0.10	0.7	0.13	**3.518**	**.016**
ROCF: copy	28.0	5.67	26.2	6.73	27.5	5.27	29.5	3.51	2.646	.050
ROCF: delayed recall	5.0	3.75	4.4	3.30	4.4	2.14	5.1	2.73	1.037	.377
Digit Span Forward	5.3	0.97	4.9	0.99	5.2	1.03	5.4	1.31	1.641	.180
Digit Span Backward	3.7	0.83	3.3	1.10	3.6	0.99	3.7	1.17	1.457	.227
Raven's Progressive Matrices	25.3	4.26	22.3	5.73	24.4	5.55	24.6	5.38	2.135	.096
Copy of figures	9.6	1.81	8.8	1.49	9.4	1.75	9.3	1.74	1.062	.366
Copy of figures with landmark	66.3	4.22	64.8	7.81	65.1	6.14	66.0	4.19	0.885	.450
Phonological Verbal Fluency	25.8	9.12	23.6	7.52	26.5	8.98	30.5	10.56	**4.186**	**.006**
Categorial Verbal Fluency	14.3	3.75	11.7	2.51	12.2	3.66	13.7	3.73	**6.146**	**.000**
Semantic–Phonological Delta (SPD)	–1.5	3.16	–2.0	2.17	–2.7	3.01	–3.3	3.52	**4.138**	**.007**
Nouns naming	28.5	1.65	28.5	1.98	27.9	2.22	28.0	2.39	1.470	.223
Stroop's test: time	43.4	28.47	62.7	57.82	49.3	26.49	51.3	32.82	2.257	.082
Stroop's test: errors	2.2	4.04	4.5	4.28	3.5	5.06	3.1	4.83	1.926	.126
MFTC: false alarms	0.6	1.73	1.2	2.55	0.6	1.62	0.8	1.88	0.955	.415
MFTC: accuracy	0.940	0.064	0.885	0.136	0.936	0.064	0.933	0.066	**3.860**	**.010**
MFTC: time of execution	78.0	36.82	86.9	45.34	85.7	33.49	76.5	32.34	1.253	.291

*Note*: Statistical significance evidenced in bold.

Abbreviations: M, mean; MFTC, Multiple Features Target Cancellation; MMSE, Mini–Mental State Examination; RAVLT, Rey's Auditory Verbal Learning Test; ROCF, Rey–Osterrieth Complex Figure; SD, standard deviation.

The post hoc comparisons showed that stable subjects obtained higher scores than fast converters on MMSE (*p* < .001); immediate recall (p = 0.002), delayed recall (*p* < .001), accuracy (*p* = .018) in recognition of RAVLT, and categorical verbal fluency (*p* = .001). When compared to intermediate converters, stable individuals obtained significantly higher scores on MMSE (*p* < .001), categorial verbal fluency (*p* = .004), and a lower SPD (*p* = .049). Finally, stable individuals obtained higher MMSE scores than slow converters (*p* = .036) and perform better only on SPD (*p* = .018).

Fast converters obtained lower scores than intermediate converters on RAVLT delayed recall (*p* = .004). When compared to slow converters, fast converters obtained lower scores on MMSE (*p* = .014); immediate recall (*p* = .014) and delayed recall (*p* < .001) on RAVLT; and phonological (*p* = .007) and categorical (*p* = .030) verbal fluency.

Finally, intermediate converters obtained lower scores than slow converters on ROCF copy (*p* = .036).

Table 2 displays the results of the multinomial regression analysis, in which the “stable” condition was set as a reference. Due to obvious collinearity effects with both categorical and phonological verbal fluency, only SPD scores entered the multinomial regression model.

**TABLE 2:** Results of the multinomial regression analyses.

		OR	95% CI		*p*
Fast	Age	1.00	0.899	1.106	.969
	Literacy	0.97	0.839	1.134	.707
	MMSE	**0.52**	**0.348**	**0.684**	**.001**
	RAVLT immediate recall	**0.90**	**0.804**	**0.989**	**.036**
	RAVLT: delayed recall	0.68	0.342	1.091	.132
	RAVLT: accuracy	0.01	0.000	1.149	.061
	Semantic–Phonological Delta	**0.80**	**0.641**	**0.955**	**.018**
Intermediate	Age	1.02	0.954	1.088	.572
	Literacy	1.05	0.960	1.158	.247
	MMSE	**0.62**	**0.479**	**0.736**	**.001**
	RAVLT immediate recall	0.96	0.903	1.017	.154
	RAVLT: delayed recall	0.98	0.741	1.304	.871
	RAVLT: accuracy	0.44	0.012	9.619	.605
	Semantic–Phonological Delta	**0.84**	**0.707**	**0.958**	**.009**
Slow	Age	1.02	0.951	1.099	.533
	Literacy	0.99	0.887	1.094	.799
	MMSE	**0.71**	**0.524**	**0.903**	**.005**
	RAVLT immediate recall	0.97	0.905	1.039	.345
	RAVLT: delayed recall	1.19	0.882	1.610	.193
	RAVLT: accuracy	0.07	0.001	3.640	.184
	Semantic–Phonological Delta	**0.78**	**0.634**	**0.897**	**.003**

*Note*: CIs were obtained after bootstrapping (1000 iterations). Statistical significance evidenced in bold. CI, confidence interval; MMSE, Mini–Mental State Examination; OR, odds ratio; RAVLT, Rey's Auditory Verbal Learning Test.

As shown, the “fast converters” category was predicted by lower scores obtained on MMSE (OR = 0.52; 95% confidence interval [CI] = 0.348–0.684; *p* = .001); RAVLT immediate recall (OR = 0.90; 95% CI = 0.804–0.989; *p* = .036); and SPD (OR = 0.80; 95% CI = 0.641–0.955; *p* = .018).

The intermediate converter category was predicted by MMSE (OR = 0.62; 95% CI = 0.479–0.736; *p* = .001) and SPD (OR = 0.84; 95% CI = 0.707–0.958; *p* = .009). Comparable results were obtained in predicting slow converters category, which was associated with lower scores on MMSE (OR = 0.71; 95% CI = 0.524–0.903; *p* = .005) and SPD (OR = 0.78; 95% CI = 0.634–0.897; *p* = .003).

## 4 DISCUSSION

Performances on neuropsychological tests have been shown to be useful to predict the progression from the prodromal phase of dementia (MCI) to overt dementia. In particular in respect of AD, both clinical evidence and neuropathological changes (that start from the medial temporal regions of the brain) indicated changes in episodic memory as among the most relevant to intercept early stages of the disease (Albert et al., 2011; Dubois et al., 2014, 2016; Fleisher et al., 2007; Gainotti et al., 2014; Loewenstein et al., 2009; Marra et al., 2015; Perri et al., 2007; Piccininni et al., 2020; Sarazin et al., 2007). Recent studies have also proposed that the analysis of lexical–semantic impairment, conducted at different levels of complexity (from the simple assessment of the number of words produced in a task of categorical fluency to fine‐grained analyses of verbal output in respect of lexical variables such as frequency and typicality) (Amieva et al., 2004; Biundo et al., 2011; Gallucci et al., 2018; Haugrud et al., 2011; Marra et al., 2021; Quaranta et al., 2016, 2019; Vita et al., 2014) may give some contribution to the identification of subjects who will progress to dementia among the ones affected by MCI. The predictive power of impairment in different cognitive domains in estimating the rate of progression from MCI to dementia has been widely explored. Nonetheless, there are no studies that explored the possibility to disentangle the role of different neuropsychological markers in predicting conversion at different time distances from the baseline. Our study aimed at this specific purpose, starting from the hypothesis that, if a marker is able to predict conversion at a distant timepoint, then we may assume that this neuropsychological marker is the expression of neuropathological changes that are at in their initial phase, and that more time is requested to reach the threshold of progression to dementia. On the opposite, if a neuropsychological marker is able to predict conversion at a near timepoint, then we may assume that neuropathology is nearer to the threshold to determine progression to dementia.

The main findings of our study show that impairment of episodic memory and lexical–semantic impairment may be putative neuropsychological predictors of progression according to the time of observation after symptoms onset.

Episodic memory measures were significantly impaired in fast converters when they were compared to stable MCI subjects, whereas the lexical–semantic impairment was the only domain significantly differentiating intermediate and slow converters from those MCI subjects who remained stable after the 6 years of follow‐up. In particular, SPD was the only marker, apart from MMSE, that differentiates stable MCI subjects and slow converters.

These findings confirm the previous evidence about the role of episodic memory impairment in predicting conversion from MCI to dementia (Gainotti et al., 2014; Marra et al., 2015; Perri et al., 2007; Sarazin et al., 2007), as a consequence of the ability of such tests in intercepting subjects who are experiencing neuropathological changes in structures belonging to the medial temporal lobe. The novelty of our findings is that the predicting power of episodic memory impairment is limited to that subjects who will progress to dementia after a relatively short time interval (“fast”), while the same measures were not able to identify subjects who progressed more slowly (“intermediate” and “slow”). Conversely, the latter categories, to which belonged subjects who developed overt dementia after a longer time interval, were predicted by a marker of lexical–semantic impairment, the SPD (Marra et al., 2021). The SPD allows us to distinguish aspects of pure loss of semantic information from the raw measure of categorical verbal fluency alone, which can also be influenced by executive aspects. The discrepancy score should provide a more sensitive detection of prodromal AD than raw semantic fluency scores, since it could factor out other aspects of verbal fluency performance that are expected to decline later. The specific decline observed only in SPD in slow decliners supports the hypothesis that a specific decline in semantic processing affects AD in the earliest stage of the disease. According to our initial hypothesis, the association between low SPD and a higher risk of being a slow decliner may indicate that it represents a marker of neuropathological changes that occur earlier than the ones leading to episodic memory impairment.

The role of semantic memory as an earlier marker than episodic long‐term memory, in detecting MCI amnesic patients prodromal to AD, has been already reported by other authors using different neuropsychological paradigms. Papp et al. (2016), using a similar measure of discrepancy between phonological and semantic verbal fluency, observed poor performances in preclinical AD several years before the onset of dementia. In their sample, subjective cognitive impaired Aβ+ subjects performed better than Aβ– on phonological verbal fluency tasks. This observation could help us to explain also our findings. In fact, a similar effect has been also found in our slow decliner group who perform better than others on phonological verbal fluency and this is probably due to a compensative effect of lexical verbal automatic output systems when the semantic verbal output, mainly based on features dependent retrieval, is defective.

The disruption of the semantic system has been also suggested in MCI prodromic to AD by paper showing a progressive reduction in generating words with low typicality (Vita et al., 2014) or more recently age of acquisition (Biundo et al., 2011) in CFT. This dysfunction has been well described within the literature as representing an early indicator of cognitive decline in AD (Amieva et al., 2004; Joubert et al., 2021; Vonk et al., 2020), but its timing of presentation with respect to long‐term memory disorders has not been explored in a longitudinal prospective way up to now.

A neuropathological explanation of the earlier impairment of semantic memory than episodic memory in AD conforms to the neuropathological staging of the disease (Braak et al., 2006). In the preclinical phase of AD, corresponding to Braak stage I, AD neurofibrillary pathology appears in the lateral transentorhinal region of the perirhinal cortex (PRC) and in the medial PRC. Thus, PRC and entorhinal cortices are affected early by tau pathology in the transentorhinal stage of AD, even before the involvement of the hippocampus (limbic stage). The involvement of these subhippocampal structures, and in particular of the PRC, could represent the neuropathological substrate involved in semantic memory.

From a clinical point of view, the relationship between semantic impairment and neuropathology of AD deserves further investigation. A correlation between t‐tau and CFT has been already described (Mirandez et al., 2017), but a wider correlation with the discrepancy scores has not been explored yet. If a significant correlation will be confirmed, this could provide a cognitive marker of neurodegeneration several years before the onset of an overt dementia permitting earlier therapeutical intervention.

### PEER REVIEW

The peer review history for this article is available at https://publons.com/publon/10.1002/brb3.3098

## Data Availability

Data sharing is not applicable to this article as no new data were created or analyzed in this study.
